# Association between physical exercise and reemployment among Chinese older adults: the mediation effect of physical health, cognitive function, and social participation

**DOI:** 10.3389/fpubh.2026.1839985

**Published:** 2026-06-09

**Authors:** Fangyi Wang, Zipeng Wu, Yu Chen, Chengyue Li, Chunhua Liu

**Affiliations:** 1 School of Sports Economics and Management, Tianjin University of Sport, Tianjin, China; 2 School of Physical Education, Xi'an University of Architecture and Technology, Xi'an, China; 3 Sports Culture Research Center, Tianjin University of Sport, Tianjin, China; 4 Research Center of Sports Humanities and Social Sciences (National Sports and Fitness Research Think Tank), Tianjin University of Sport, Tianjin, China; 5 Human Resources Department, Tianjin University of Sport, Tianjin, China

**Keywords:** active aging, mediation effect, older adults, physical exercise, reemployment, social participation

## Abstract

**Objective:**

This study aims to systematically investigate the effects, mechanisms, and demographic heterogeneity of physical exercise on reemployment among older adults. To address the gaps in current studies, i.e., the lack of exploration of mediating mechanisms and the scarcity of large-sample empirical evidence, this paper provides empirical evidence and practical guidance for activating the human resources of older adults and advancing active aging strategies.

**Methods:**

Data were derived from the 2023 China Longitudinal Aging Social Survey, yielding a sample of 4,597 retired individuals aged 60 and older with complete data. The study treated reemployment as the dependent variable and physical exercise behavior as the primary independent variable, with physical health, cognitive function, and social participation selected as mediating variables. Control variables included factors related to individual and household sociodemographic characteristics. A binary logistic regression model was used to analyze main effects, and the propensity score matching method was employed to address potential endogeneity. Robustness tests were conducted using multiple methods, and the mediating effects were examined using the bias-corrected Bootstrap method, while heterogeneity analysis was also performed.

**Results:**

The baseline regression results revealed that physical exercise has a significant positive effect on reemployment among Chinese older adults. This effect remained stable and reliable after undergoing several robustness checks, including propensity score matching and other regression models. Tests of the mediating effects confirmed that physical exercise could promote reemployment among older adults through three pathways: first, by improving physical health, with an indirect effect accounting for approximately 10.3% of the total effect; second, by enhancing cognitive functions, with an indirect effect accounting for approximately 4.9% of the total effect; third, by increasing social participation, with an indirect effect accounting for approximately 11.3% of the total effect, which serves as the core mediating pathway. Heterogeneity analysis revealed significant group differences in this promotional effect. The promotional effect of physical exercise was particularly pronounced among older adults in rural areas, the eastern region, those aged 60–70 years, those with a junior middle school education or above, and those who were ordinary workers, clerks, or other before retirement.

**Conclusion:**

Physical exercise was and significantly positively associated with reemployment among older adults. This association may operate through several parallel pathways: physical exercise was associated with higher reemployment likelihood partially via improving physical health, enhancing cognitive functions, and increasing social participation, with social participation serving as the most critical mediating pathway.

## 1 Introduction

China is rapidly transitioning into a deeply aged society. According to 2025 data from the National Bureau of Statistics, the proportion of the population aged 60 and above surpassed 22% by the end of 2024 (1), exceeding the United Nations benchmark for a moderately aged society (20%). Against this backdrop, developing and utilizing the “silver workforce” in the labor market has become a crucial pathway to alleviate labor shortages and tap into the demographic dividend of the older adults population. The Opinions of the Central Committee of the Communist Party of China and the State Council on Strengthening Aging Work in the New Era designate sports as a core measure to address aging, emphasizing the extension of older adults’ social participation through health interventions (2). The General Administration of Sport of China’s Opinions on Further Strengthening Sports for the Older Adults in the New Era proposes advancing older adults sports development in line with emerging trends and leveraging sports’ positive role in addressing population aging (3). This aims to enhance health capital and social participation capacity in older adults through age-friendly sports services. How physical exercise can extend the occupational lifecycle of older adults has become a critical issue in tackling aging challenges.

As a vital practice of active aging, reemployment among older adults not only serves as a key way to sustain their social value but also provides an effective means to alleviate the pressure of older adults’ care and optimize the labor force structure. Physical exercise, serving as a bridge connecting physical and mental health with social participation, is increasingly drawing attention from both policymakers and scholars for its role in promoting reemployment among older adults. The 14th Five-Year Plan for National Aging Development and Older adults Care Services explicitly proposes building an age-friendly society, guiding older adults to embrace proactive health concepts, encouraging their full participation in economic and social development, and emphasizing the need to improve health support systems for the older adults, expand health service resources, and develop home-based medical services (4). This policy direction not only requires society to create a supportive environment for older adults’ reemployment but also emphasizes enhancing their employability through health interventions. Current research has revealed that regular physical activity can lay the physical foundation for older adults to extend their professional careers by delaying muscle atrophy and enhancing memory and executive function (5, 6). Participation in group sports activities can serve as a channel for older adults to access employment information and build social trust (7). Although previous studies have explored the association between physical exercise and reemployment among older adults, research on the mediating effects of physical exercise in this context remains scarce, and there is a lack of large-scale empirical evidence. Therefore, this study utilized data from the 2023 China Longitudinal Aging Social Survey (CLASS) to systematically examine the comprehensive impact mechanism of physical exercise on the reemployment of older adults through logistic regression and mediation analysis. It specifically tested the mediating roles of physical health, cognitive ability, and social participation. The findings aim to provide empirical support for refining policies integrating physical fitness and health, developing human resources among older adults, and advancing the goal of active aging through meaningful engagement in later life.

## 2 Literature review and research hypotheses

### 2.1 Association between physical exercise and reemployment among older adults

Physical exercise was the systematic process of physical activity undertaken by individuals during leisure time or self-scheduled periods, employing diverse sports methods and strategies to enhance physical fitness, promote mental and physical health, or achieve other specific developmental goals (8, 9). For older adults, physical exercise held profound significance extending far beyond mere physical conditioning. Physical exercise not only effectively delayed physiological decline and enhanced vitality among older adults but also significantly improved their mental health and subjective well-being by expanding social networks and increasing social integration (10–12). This comprehensive enhancement of physical and mental states, coupled with accumulated social capital, formed a crucial foundation for older adults to reintegrate into society and experience a “second spring.” It elevated their willingness to participate in social activities and enhanced their capacity for reemployment. First, health served as the fundamental prerequisite for older adults to sustain their participation in social labor. Some studies emphasized that during the process of active aging, physical exercise can enhance older adults’ physical and mental health levels while strengthening their capacity for social participation (13, 14). This, in turn, continuously promoted their social adaptability and occupational continuity. This perspective was empirically supported by Abdine et al. (15), where regular physical activity was shown to significantly reduce risks such as cognitive decline in older adults while maintaining the physical and mental capacities required for work. Second, the skills and experience gained through physical exercise itself become valuable capital for career transitions among older adults. Hanson et al. (16) noted that older adults with sports expertise or experience can transition into new professional roles such as coaches, referees, trainers, or community instructors, thereby extending the value of their skills. More importantly, the universal competencies cultivated through physical exercise, such as communication, organization, team collaboration, and a positive mindset and adaptability, can effectively enhance older adults’ competitiveness across broader employment sectors and meet diverse job requirements. Finally, the process of deeply engaging in sports activities itself constituted a form of positive social role-shaping and value creation. Jenkins et al. (17) found that older adults contributed valuable social capital and stability through sports volunteering, knowledge transfer, and participation in community sports activities. Moreover, these activities invisibly enhanced their social recognition and expanded their interpersonal networks, directly strengthening their social capital and adaptability in seeking and adjusting to reemployment opportunities. In summary, physical exercise built a bridge for older adults to re-enter or sustain participation in the labor market. Based on this, this study proposed hypothesis 1:

*H*1: Participation in physical exercise has a positive impact on reemployment among older adults.

### 2.2 Association between physical health, physical exercise, and reemployment among older adults

Physical exercise has significant and multifaceted positive effects on improving and maintaining the physical health of older adults. Research based on large-scale longitudinal data has revealed that, despite complex dynamic variations in these health benefits across age, time periods, and generations, exercise generally delivers substantial physical health advantages for older adults. This underscored the critical role of sustained physical activity in achieving healthy aging (18). Physical exercise effectively enhanced physical fitness, managed chronic diseases, optimized body functions, and delayed age-related functional decline (19, 20). An intervention study by Takayanagi et al. (21) further provides empirical support: increasing daily low-intensity physical activity significantly improves health indicators among older adults by enhancing cognitive function, stabilizing mood, and optimizing sleep quality. Moreover, activity duration shows a positive correlation with overall health levels.

Notably, the physical health improvements resulting from exercise not only enhance the quality of life for older adults but also directly strengthen their physical functions, energy reserves, and work endurance. This lays a solid health foundation for older adults to actively integrate into society and participate in various social activities, including reemployment. Among individual characteristics, physical health status exerts a significant positive influence on reemployment among older adults (22). Healthy older adults possess greater energy and mobility, enabling them to better navigate the challenges of reemployment. This mechanism is particularly pronounced among specific groups when household labor outflows occur, with younger, physically fit rural male older adults experiencing significantly higher employment probabilities (23). Research on social participation in older adults further corroborates the bidirectional reinforcing relationship between health and employment (24). Full-time employment in older age not only relies on physical health but also, as an active form of social participation, significantly enhances cognitive function and activities of daily living, thereby creating a virtuous cycle where health supports employment, and employment promotes health. In summary, physical exercise enhances health for older adults by improving physical fitness, managing chronic diseases, and delaying functional decline. Improved health status directly increases capacity for social participation and reemployment among older adults. Based on this, this study proposed hypothesis 2:

*H*2: Physical health mediated the impact of physical exercise on reemployment among older adults.

### 2.3 Association between cognitive functions, physical exercise, and reemployment among older adults

Physical exercise has gained academic attention in recent years as an effective intervention for enhancing cognitive functions in older adults. Physical exercise can significantly enhance response inhibition in older adults by regulating attention resource allocation and changes in frontal lobe gray matter volume, providing neurobiological evidence for cognitive function protection (25). Callow et al. (26) found that a single session of moderate-intensity aerobic exercise reduced cognitive interference by improving hippocampal-dependent memory discrimination in older adults. A review by Klimova et al. (27) on older adults in developed countries indicated that diverse physical activities, including gymnastics, enhance cognitive function through neuroplasticity mechanisms. Another systematic review reported that various exercise types, such as aerobic, resistance, and multi-component training, improve overall cognitive function in older adults. The most significant effects were observed with moderate-intensity interventions, showing significant improvements (28).

Notably, the maintenance and enhancement of cognitive abilities facilitated by physical exercise not only formed a crucial foundation for older adults to tackle complex tasks, but also directly support their capacity to re-enter the workforce and extend their career lifecycles. This established a vital cognitive foundation for older adults to continue contributing societal value and realizing their professional potential. Systematic reviews indicate that targeted cognitive training (e.g., memory and executive function training) significantly improves cognitive levels in older adults. This mechanism operates by enhancing neuroplasticity and optimizing core functions such as working memory, thereby directly boosting older adults’ capacity to handle complex occupational tasks (29). This conclusion resonates with Delaporte’s (30) empirical study of Chilean older adults: those who remained employed demonstrated superior memory and executive function due to the sustained cognitive stimulation from occupational activities. Conversely, the maintenance of cognitive abilities further enhanced their adaptability in the workplace, ultimately creating a positive feedback loop. Cognitive health served as the fundamental prerequisite for older adults to develop a willingness to re-enter the workforce (31). Higher cognitive levels enable them to more effectively coordinate work-life balance, alleviate role transition stress, and thereby enhance their propensity for reemployment driven by both economic necessity and self-actualization motivation. In summary, physical exercise effectively enhances cognitive abilities in older adults through multiple mechanisms, including boosting neuroplasticity, optimizing attention regulation, and improving hippocampal function. Enhanced cognitive abilities not only strengthen older adults’ capacity to learn new skills but also optimize their decision-making efficiency, enabling them to maintain competitiveness in a rapidly evolving reemployment environment characterized by rapid knowledge updates. Based on this, this study proposed hypothesis 3:

*H*3: Cognitive functions mediated the impact of physical exercise on reemployment among older adults.

### 2.4 Association between social participation, physical exercise, and reemployment among older adults

Physical exercise serves as an effective vehicle for enhancing social connections among older adults, with its promotional effects on social participation supported by multidimensional research. Participation in sports significantly accumulated individual social capital by enhancing interpersonal interaction skills and improving social evaluations, while also expanding social capital through channels such as political engagement and organizational involvement (32). Physical exercise effectively promotes their social integration through dual pathways: improving subjective health (enhancing physical and mental well-being) and expanding objective social networks (broadening social circles) (33). From a health promotion perspective, physical exercise significantly improved the probability of social participation among older adults through direct effects and the mediating effects of health indicators. Urban–rural disparities, age, and marital status exerted moderating roles in this process, further highlighting the universality and importance of physical exercise in building social networks for older adults (34). Research by Nau et al. (35) on vulnerable older adults emphasized that precisely matching exercise interests, optimizing venue accessibility, and fostering a supportive fitness environment are key strategies to help older adults overcome social anxiety and social limitations.

Physical exercise effectively enhanced social participation for older adults, serving not only as a key driver for building an active aging society but also directly supporting the activation and improvement of their reemployment capabilities. This provided a crucial social foundation for their continued integration into society and contribution of value. Older adults could make positive contributions across multiple dimensions, including health, consumption, and labor, through sustained social engagement such as community service and volunteer activities (36). Such participation not only helped maintain their physical functions but also reinforced their sense of self-worth through specific role-playing, thereby laying the groundwork for reemployment capabilities. A cross-national comparative study reported that the external social recognition gained by older adults through social participation can effectively reduce barriers to their inclusion in the labor market while enhancing employers’ willingness to hire them (37). Socially active and positively minded older adults are more likely to maintain midlife behavioral patterns, such as preserving work continuity and strengthening social interactions, which significantly increases their employment prospects. Central to this process was how social engagement continuously exercises and preserves cognitive flexibility and problem-solving abilities in older adults (38). In summary, physical exercise enhances social participation for older adults by multiple pathways: improving interpersonal interaction skills, enhancing physical and mental health, and expanding social networks. Increased social participation not only strengthens older adults’ sense of self-worth and physical functioning but also exercises their cognitive flexibility, helping them activate reemployment potential and maintain professional vitality. Based on this, this study proposed hypothesis 4:

*H*4: Social participation mediated the impact of physical exercise on reemployment among older adults.

**Figure 1:**
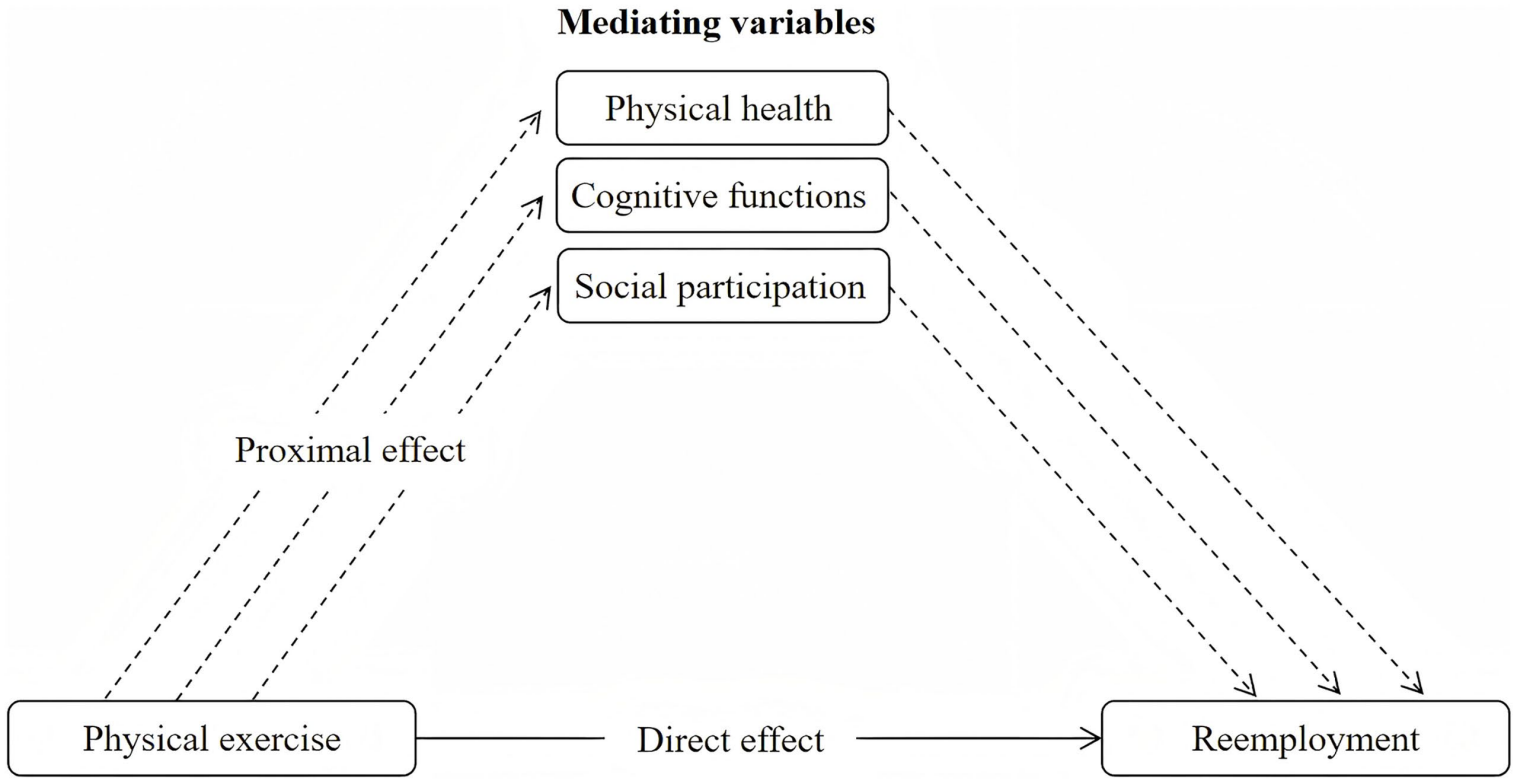
Theoretical analysis framework diagram.

In summary, the relationship diagram between physical exercise and reemployment among older adults was as follows in Figure 1.

## 3 Methods

### 3.1 Data sources

Data for this study were derived from CLASS2023. Designed by the Institute of Gerontology at Renmin University of China and implemented by the China Survey and Data Center, this project constituted a nationwide longitudinal survey of individuals aged 60 and above. The survey encompasses multidimensional information, including physical health status, socioeconomic conditions, retirement planning, social engagement, mental health, and family background among older adults. The CLASS survey employed a stratified multistage probability sampling method, covering 28 provinces (municipalities and autonomous regions) nationwide. Given that the latest 2023 survey included supplementary questions on retirement and reemployment status, this study specifically selected data from that year for analysis. This study treated reemployment among older adults as the dependent variable. From an initial sample of 11,671 individuals, retired older adults who selected “Yes” to question C6 (“Have you retired?”) in the questionnaire were identified as research subjects. After data cleaning, 4,597 valid samples were ultimately obtained.

### 3.2 Variables

#### 3.2.1 Dependent variable

This study used reemployment among older adults as the dependent variable. Reemployment was defined as the situation where older adults who had completed retirement procedures subsequently engaged in income-generating work or activities. Based on previous research (39, 40), question C1 from the CLASS2023 questionnaire (“What is your current status regarding income-generating work or activities?”) was selected to construct the variable. A binary classification method was applied. Individuals who responded “Almost every day,” “At least once a week,” “At least once a month,” or “Several times a year” were classified as reemployed and coded as 1. Those who responded “Not participating” were classified as not reemployed and coded as 0.

#### 3.2.2 Core explanatory variable

This study used physical exercise behavior among older adults as the core explanatory variable. Using the question G2 (“How often do you engage in physical exercise?”) from the CLASS2023 questionnaire to construct the variable. A binary classification approach was applied: participants who responded “less than once a month” were considered to rarely engage in physical exercise and coded as 0; all other responses indicated physical exercise behavior and were coded as 1. This classification criterion was selected with reference to the commonly used standards in domestic and international older adults health research, which generally define “basic exercise habit formation” as participating in physical activity at least once a month (41, 42). According to the conceptual definition of physical activity, engaging in physical activity less than once per month is considered occasional rather than regular (41).

#### 3.2.3 Mediating variables

This study used physical health, cognitive functions, and social participation as mediating variables. Physical health: Self-rated physical health reflects individuals’ subjective perceptions of their physiological functioning, chronic disease burden, and adaptive capacity in daily life, serving as an indicator of physical health. Question B1: “How would you describe your current physical health?” was quantified on a 1–5 scale (1 = very unhealthy, 5 = very healthy), with higher scores indicating better health. Cognitive functions: Following Wang et al. (43), cognitive function was measured using the word-based test score from a question group of E1 (“I will ask you a few questions; please answer to the best of your ability”). Scores covered four domains, including spatial–temporal orientation (5 points), short-term memory (3 points), calculation ability (5 points), and delayed memory (3 points). The total score was 16 points, with higher scores indicating stronger cognitive functions. Social participation: Following a previous study (44), question D14 (“In the past year, how often did you participate in the following activities?”) was used to measure social participation levels among older adults. This question included seven sub-items: participating in community patrols, caring for other older adults or children, environmental protection, mediating disputes, providing companionship, volunteering requiring specialized skills, and caring for the education of the next generation. Each subitem was scored on a scale of 1 to 5, with a maximum total score of 35 points. Higher scores indicated a greater level of social participation.

#### 3.2.4 Control variables

To enhance the reliability of the research findings, this study selected control variables from the dimensions of individual characteristics of older adults and family-societal characteristics, building upon previous research (39, 45). Individual characteristic variables included sex (male = 1, female = 0), age (continuous variable), educational level (junior middle school or above = 1, other = 0), presence of chronic disease (yes = 1, no = 0), and pre-retirement occupational status (entry-level manager or above = 1, ordinary worker, clerk, or other = 0). Family and social characteristic variables included household registration type (non-agricultural = 1, agricultural = 0), marital status (married with a spouse = 1, widowed, divorced, and unmarried = 0), number of offspring (continuous variable), and availability of exercise facilities in the community (available = 1, unavailable = 0). Descriptive statistics for these variables were detailed in Table 1.

**Table 1:** Descriptive statistics of variables (*N* = 4,597).

Category	Variables	Code	Min	Max	Mean	SD
Dependent variable	Reemployment	Yes = 1, No = 0	0	1	0.072	0.258
Independent variable	Physical exercise	Yes = 1, No = 0	0	1	0.559	0.497
Mediating variables	Physical health	Continuous (1–5 points)	1	5	3.67	0.84
	Cognitive functions	Continuous (1–16 points)	1	16	14.333	2.193
	Social participation	Continuous (1–35 points)	1	35	9.49	4.256
Control variables of individual characteristics	Sex	Male = 1, Female = 0	0	1	0.521	0.5
	Age	Continuous	60	96	70.508	5.653
	Educational level	Junior middle school or above = 1, Other = 0	0	1	0.702	0.457
	Chronic disease	Yes = 1, No = 0	0	1	0.227	0.419
	Occupation before retirement	Entry-level manager or above = 1, Ordinary worker, clerk, or other = 0	0	1	0.13	0.336
Control variables of family-societal characteristics	Household registration	Non-agricultural = 1, Agricultural = 0	0	1	0.886	0.317
	Marital status	Married with a spouse = 1, Widowed, divorced, and unmarried = 0	0	1	0.888	0.316
	Number of offspring	Continuous	0	9	1.644	0.945
	Availability of exercise facilities in community	Yes = 1, No = 0	0	1	0.175	0.38

Data sourced from 2023 CLASS. Min is the minimum value, Max is the maximum value, and SD is the standard deviation.

### 3.3 Model construction

#### 3.3.1 Main effects model: logistic regression model

To estimate the direct impact of physical exercise on reemployment among older adults (H1) and construct the following logistic regression model (Equation 1):


$$\text{Logit}(P(\text{Reemploymen}t_{i}=1))=\beta_{0}+\beta_{1}\text{Exercise}i_{i}+\gamma X_{\mathrm{ij}}+\mu_{i}$$ (1)

*Reemployment* was the dependent variable, *Exercise* was the core explanatory variable, *β*_0_ was the constant term, *β*_1_ was the coefficient of the core explanatory variable, *X*_ij_ were the control variables, *γ* was the coefficient vector of *X*_ij_, and *μ*_i_ was the random error. The model employed maximum likelihood estimation to interpret the direction and degree of influence between physical exercise and reemployment probability by examining odds ratios (ORs) and marginal effects. If *β*_1_ is significantly positive, it supports hypothesis H1.

#### 3.3.2 Mediation effect model: bias-corrected bootstrap method

To verify the mediating effects of physical health, cognitive function, and social participation (H2-H4), this study employed a bias-corrected Bootstrap method to construct mediation effect models. The detailed steps were as follows:

Step 1: Estimate the impact of physical exercise among older adults on the mediating variables. Linear regression models were fitted with physical health, cognitive function, and social participation as dependent variables, respectively (Equation 2):


$$\text{Mediato}r_{i}=\alpha_{0}+\text{αExercise}i_{i}+\gamma X_{\mathrm{ij}}+\mu_{i}$$ (2)

Where *Mediator*_i_ represented three mediating variables in sequence, *α*_0_ was the constant term, *α* was the coefficient of Exercise’s effect on the mediating variables, *X*_ij_ represented the control variables, *γ* represented the coefficients of control variables, and *μ*_i_ was the random error. If the coefficient *α* is significant, it indicates that physical exercise among older adults exerts a direct effect on the mediating variables.

Step 2: Incorporate mediating variables into the final main effects model (Equation 3):


$$\begin{array}{c} \text{Logit}(P(\text{Reemploymen}t_{i}=1))=\beta_{0}^{\prime }+\beta_{1}^{\prime }\text{Exercise}i_{i}\\ +\text{δMediato}r_{i}+\gamma X_{\mathrm{ij}}+\mu_{i}^{\prime } \end{array}$$ (3)

Where $\beta_{0}^{\prime }$ was the new constant term, $\beta_{1}^{\prime }$ was the coefficient representing the direct effect of Exercise on reemployment, *δ* was the coefficient representing the effect of Mediatori on reemployment, *X*_ij_ was the control variable, *γ* represented the coefficients of control variables, and $\mu_{i}^{\prime }$ was the new random error. If the mediating variable coefficient *δ* is significant and the absolute value of $\beta_{1}^{\prime }$ is smaller than that of *β*_1_ in the main effects model, this indicates the presence of a mediating effect. Based on whether $\beta_{1}^{\prime }$ is significant, the mediating effect can be further determined as partial mediation or full mediation.

## 4 Results

### 4.1 Baseline logistic regression

This study utilized a binary logistic regression model for baseline analysis and verified the robustness of results through the stepwise inclusion of control variables. As shown in Table 2, Model (1) controlled only for physical activity and provincial fixed effects among older adults. Model (2) added personal characteristic variables, including sex, age, educational level, presence of chronic diseases, and occupation before retirement. Model (3) further incorporated social and family characteristic variables such as household registration type, marital status, number of offspring, and availability of exercise facilities in community. The baseline regression analysis revealed that the coefficients for physical exercise among older adults were significantly positive in all three models (*p* < 0.001), gradually increasing from 0.505 in Model (1) to 0.690 in Model (3). This suggested that physical exercise had a significant positive impact on reemployment among older adults, with this effect becoming more pronounced as additional control variables were incorporated. This reflected robust statistical results and a well-designed model.

**Table 2:** Baseline logistic regression of the impact of physical exercise on reemployment among Chinese older adults.

Variables	Model (1)	Model (2)	Model (3)
	Reemployment	Reemployment	Reemployment
Physical exercise	0.505 (0.121)^***^	0.514 (0.134)^***^	0.690 (0.140)^***^
Sex		0.352 (0.125)^**^	0.348 (0.128)^**^
Age		−0.115 (0.013)^***^	−0.128 (0.014)^***^
Educational level		−0.856 (0.137)^***^	−0.611 (0.143)^***^
Chronic disease		0.047 (0.149)	−0.115 (0.156)
Occupation before retirement		0.592 (0.157)^***^	0.676 (0.161)^***^
Household registration			−1.504 (0.153)^***^
Marital status			−0.534 (0.197)^**^
Number of offspring			0.180 (0.070)^**^
Availability of exercise facilities in the community			−0.076 (0.169)
Intercept	−2.868 (0.098)^***^	5.278 (0.967)^***^	7.315 (1.053)^***^
Provincial fixed effects	Yes	Yes	Yes
Observation	4,597	4,597	4,597
*R* ^2^	0.107	0.176	0.235

Values in parentheses represent robust standard errors. * represents *p* < 0.05, ** represents *p* < 0.01, and *** represents *p* < 0.001.

From the perspective of control variables, sex had a significant positive effect on reemployment among older adults, indicating that male older adults had a significantly greater probability of reemployment than female older adults. This may be related to the inertia of male economic participation within traditional family divisions of labor. Younger older adults had a greater probability of reemployment, with physical strength and energy advantages likely being important factors. Educational level showed a significant negative correlation with reemployment, suggesting that older adults with higher education levels may have more secure economic foundations after retirement and thus lower reemployment needs. This finding was contrary to the general expectation that higher education levels enhance employ ability, which might be due to the fact that highly educated older adults in China were more likely to enjoy generous pension benefits and had stronger financial independence, leading to lower motivation for economic reemployment. In addition, they might prefer non-economic social participation activities such as volunteer work rather than paid employment. The occupation before retirement had a significant positive impact on reemployment, indicating that older adults with professional skills or accumulated resources are more likely to secure new employment. Additionally, non-agricultural household registration and marital status were associated with lower reemployment probabilities, possibly because these groups enjoy more comprehensive social security or family support. A greater number of offspring was linked to higher reemployment probabilities, which may relate to increased demand for family financial support. The presence of chronic diseases and the availability of exercise facilities in community had insignificant effects. The insignificant effect of community exercise facilities might be attributed to the low coverage rate of such facilities in the sample (only 17.5% of respondents reported having exercise facilities in their communities), and most older adults who exercise regularly chose public spaces such as parks and squares rather than community facilities. In addition, the variable only measured the availability of facilities but not their quality, accessibility and usage frequency, which might also lead to insignificant results. In summary, H1 was confirmed.

### 4.2 Robustness test

To ensure the reliability of the baseline regression results, this study conducted four robustness tests: (1) overfitting test, which estimated the model by simultaneously incorporating all three mediating variables into the baseline model; (2) replacing the regression model, which used a Probit model instead of a binary logistic regression model to account for the binary nature of the dependent variable. (3) Sample redefinition: following Zhou et al. (46), the sample was focused on the primary group of re-employed older adults, restricted to younger older adults aged 60 to 70, and analyzed using the Probit model. (4) Continuous treatment: A three-category approach of frequency was applied. Frequency of less than once a month was coded as 0. Frequency of one time or more a month but less than once a day was coded as 1. Frequency of once or more a day was coded as 2. Physical exercise was treated as a continuous variable in the model. Table 3 shows that the coefficient of physical exercise among older adults consistently maintained significant positive directions (*p* < 0.05) in all three robustness tests.

**Table 3:** Robustness test results.

Variables	Overfitting	Probit model	Older adults aged 60–70 years	Continuous treatment^#^
Physical exercise	0.566 (0.146)^***^	0.300 (0.064)^***^	0.371 (0.085)^***^	0.189 (0.084)*
Control variables	Yes	Yes	Yes	Yes
Intercept	0.352 (1.369)^**^	3.615 (0.505)^***^	3.734 (0.932)^***^	8.081 (1.003)***
Provincial fixed effects	Yes	Yes	Yes	Yes
Observation	4,597	4,597	2,482	4,597
*R* ^2^	0.196	0.154	0.131	0.120

Values in parentheses represent robust standard errors. * represents *p* < 0.05, ** represents *p* < 0.01, and *** represents *p* < 0.001. ^#^ A three-category approach of frequency was applied. Frequency of less than once a month was coded as 0. Frequency of one time or more a month but less than once a day was coded as 1. Frequency of once or more a day was coded as 2. Physical exercise was treated as a continuous variable in the model.

### 4.3 Treatment of endogeneity

Given that baseline regression analysis may face endogeneity problems caused by self-selection. For example, physical exercise can promote reemployment among older adults, but those with better health may inherently be more inclined to maintain exercise habits. To ensure the reliability of findings, this study adopted a propensity score matching (PSM) model to address potential endogeneity. As shown in Table 4, before matching, significant differences existed between exercising and non-exercising older adults in covariates such as age, education level, and presence of chronic diseases. After matching, standardized bias for most variables decreased notably, with absolute standardized bias values below 20% (most below 10%), and all variables yielded *p*-values greater than 0.05. This indicates that the two groups no longer exhibit significant differences in covariates, effectively satisfying the balance requirement. This supports the robustness of the baseline regression results, confirming that the sample passed the balance test.

**Table 4:** Sample balance test results.

Variables	Matching	Exercise	No exercise	Standardized bias (%)	Decreases of standardized bias (%)	*T* value	*p* value
Sex	Before	0.51	0.53	−4.02	8.45	−1.35	0.078
	After	0.51	0.53	−3.68		−0.86	0.389
Age	Before	70.12	72.03	−32.95	53.11	−7.42	<0.001
	After	70.12	71.00	−15.45		−5.15	0.081
Educational level	Before	0.70	0.61	19.14	86.52	4.40	<0.001
	After	0.70	0.71	−2.58		−0.87	0.386
Chronic disease	Before	0.16	0.31	−36.69	31.09	−12.13	<0.001
	After	0.16	0.26	−15.28		−5.63	0.168
Occupation before retirement	Before	0.14	0.17	−8.42	48.34	−1.93	0.045
	After	0.14	0.12	4.35		1.46	0.144
Household registration	Before	0.93	0.84	28.66	55.65	9.42	<0.001
	After	0.93	0.88	12.71		4.09	0.263
Marital status	Before	0.90	0.84	17.89	45.56	3.96	0.001
	After	0.90	0.87	9.74		3.24	0.092
Number of offspring	Before	1.65	1.79	−15.44	87.18	−3.55	<0.001
	After	1.65	1.64	1.98		0.33	0.744
Availability of exercise facilities in the community	Before	0.20	0.14	16.43	90.03	5.56	<0.001
	After	0.20	0.21	−1.64		−0.38	0.703

The sample balance test results are based on the matching outcomes from the nearest neighbor matching method.

To ensure the robustness of the results and avoid reliance on a single matching method, this study employed three matching methods: nearest-neighbor matching, radius matching, and kernel matching. As shown in Table 5, the average treatment effects (ATTs) were significantly positive at the 1% level across all three methods, with specific values of 0.034, 0.019, and 0.024, respectively. Overall, after validation using multiple matching methods, the conclusions of the baseline regression remained valid and robust. It should be emphasized that propensity score matching only alleviates the selection bias caused by observable covariates, and cannot eliminate the influence of unobservable confounding variables. Therefore, after controlling for the effects of observable confounding variables in the sample, the relationship between physical exercise and reemployment among older adults was still a stable and significant correlation, rather than an absolute causal relationship.

**Table 5:** ATT of propensity score matching.

Matching methods	Treatment	Control	ATT	Standard error	*T* value
Nearest-neighbor	0.079	0.045	0.034^***^	0.006	5.90
Radius	0.079	0.060	0.019^***^	0.007	2.71
Kernel	0.079	0.055	0.024^***^	0.012	3.88

ATT is the average treatment effect. * represents *p* < 0.05, ** represents *p* < 0.01, and *** represents *p* < 0.001.

### 4.4 Mechanism analysis

To further explore the mechanisms through which physical exercise among older adults influences reemployment behavior, this study employed the bias-corrected Bootstrap method (5,000 resamples) to systematically analyze the mediating pathways of physical health (M1), cognitive functions (M2), and social participation (M3). As shown in Table 6:

**Table 6:** Analysis of the mediation mechanism in the impact of physical exercise on reemployment among older adults.

Mediating variables	Category	Result	Conclusion
Physical health	Pathway	X → M1 → Y	Partial mediation effect
	Coefficient of pathway a	0.132^**^	
	Coefficient of pathway b	0.031^**^	
	Indirect effect of a × b	0.004	
	Standard error	0.002	
	95% confidence interval of mediating effect	[0.003, 0.012]	
	Coefficient of direct effect c’	0.029^**^	
	Coefficient of total effect c	0.039^**^	
	Proportion of the mediating effect	10.3%	
	Control variables	Yes	
Cognitive functions	Pathway	X → M2 → Y	Partial mediation effect
	Coefficient of pathway a	0.410^**^	
	Coefficient of pathway b	0.005^**^	
	Indirect effect of a × b	0.002	
	Standard error	0.001	
	95% confidence interval of mediating effect	[0.001, 0.006]	
	Coefficient of direct effect c’	0.029^**^	
	Coefficient of total effect c	0.039^**^	
	Proportion of the mediating effect	4.9%	
	Control variables	Yes	
Social participation	Pathway	X → M3 → Y	Partial mediation effect
	Coefficient of pathway a	0.940^**^	
	Coefficient of pathway b	0.005^**^	
	Indirect effect of a × b	0.004	
	Standard error	0.003	
	95% confidence interval of mediating effect	[0.003, 0.014]	
	Coefficient of direct effect c’	0.029^**^	
	Coefficient of total effect c	0.039^**^	
	Proportion of the mediating effect	11.3%	
	Control variables	Yes	

“a” represents the pathway through which the main explanatory variable influences the mediating variable; “b” represents the pathway through which the mediating variable influences the dependent variable; “c’” represents the independent effect of the main explanatory variable on the dependent variable after controlling for the mediating variable; and “c” represents the overall effect of the main explanatory variable on the dependent variable. M1 represents the physical health, M2 represents the cognitive functions, and M3 represents the social participation. * represents *p* < 0.05, ** represents *p* < 0.01, and *** represents *p* < 0.001.

First, the mediating effect of physical health was significant. The pathway analysis revealed that physical exercise had a significant positive predictive effect on physical health among older adults (a = 0.132, *p* < 0.01), and physical health also had a significant positive predictive effect on reemployment behavior (b = 0.031, *p* < 0.01). The total effect of physical exercise on reemployment was 0.039 (*p* < 0.01). After controlling for health variables, the direct effect remained significant (c’ = 0.029, *p* < 0.01), indicating the presence of a partial mediating relationship. Bootstrap tests further confirmed that the indirect effect of this pathway was 0.004 (95% CI: 0.003, 0.012), accounting for 10.3% of the total effect. This result validated that improving health was a key pathway for physical exercise to promote reemployment.

Second, the mediating role of cognitive functions was confirmed. Physical exercise had a significant positive predictive effect on cognitive functions (a = 0.410, *p* < 0.05), indicating that exercise helps delay cognitive decline; simultaneously, cognitive functions also had a significant positive impact on reemployment (b = 0.005, *p* < 0.01), highlighting the importance of cognitive functions in labor market participation among older adults. The indirect effect of this pathway was 0.002 (95% CI: 0.001, 0.006), accounting for 4.9% of the total effect, indicating that physical exercise promotes reemployment by enhancing cognitive functions. Although this mechanism was secondary, it was statistically significant.

Finally, the mediating effect of social participation was the most pronounced. Physical exercise had a very strong positive predictive effect on the level of social participation (a = 0.940, *p* < 0.01), reflecting its central role in expanding social networks; simultaneously, the level of social participation had a significant positive impact on reemployment (b = 0.005, *p* < 0.01), confirming the promotional role of social connections in reemployment among older adults. The indirect effect of this pathway was 0.004 (95% CI: 0.003, 0.014), accounting for 11.3% of the total effect, making it the primary mechanism among the three pathways. In summary, H2, H3, and H4 were all confirmed, indicating that participation in physical exercise among older adults can improve their physical health, enhance cognitive function, and increase social participation, thereby effectively promoting their reintegration into the workforce.

### 4.5 Heterogeneity analysis

Older adults exhibited significant differences in living environments, cultural backgrounds, and social characteristics, and these differences profoundly influenced their physical exercise habits and reemployment. Based on this, this study examined the overall impact of physical exercise on reemployment among older adults and further analyzed the heterogeneity of its effects across five dimensions: urban–rural, area, age category, educational level, and occupation before retirement (Tables 7, 8).

**Table 7:** Results of heterogeneity analysis (1).

Variables	Residence		Area	
	Urban	Rural	East	Central-west
Physical exercise	0.559 (0.161)^***^	0.762 (0.280)^**^	0.867 (0.214)^***^	0.373 (0.19)^*^
Control variables	Yes	Yes	Yes	Yes
Intercept	5.222 (1.209)^***^	8.758 (2.251)^***^	4.538 (1.586)^**^	9.278 (1.454)^***^
Provincial fixed effects	Yes	Yes	Yes	Yes
Observation	4,075	522	3,057	1,540
*R* ^2^	0.163	0.298	0.123	0.187

Values in parentheses represent robust standard errors. * represents *p* < 0.05, ** represents *p* < 0.01, and *** represents *p* < 0.001.

**Table 8:** Results of heterogeneity analysis (2).

Variables	Age category		Educational level		Occupation before retirement	
	60–70 years	71 years and above	Junior middle school or above	Other	Entry-level manager or above	Ordinary worker, clerk, or other
Physical exercise	0.742^***^ (0.175)	0.593^*^ (0.237)	0.703^***^ (0.176)	0.601^**^ (0.232)	0.516 (0.327)	0.725^***^ (0.156)
Control variables	Yes	Yes	Yes	Yes	Yes	Yes
Intercept	7.783^***^ (1.834)	9.880^***^ (2.916)	6.851^***^ (1.32)	6.475^***^ (1.692)	9.357^***^ (2.647)	7.053^***^ (1.159)
Provincial fixed effects	Yes	Yes	Yes	Yes	Yes	Yes
Observation	2,482	2,115	3,228	1,369	593	4,004
*R* ^2^	0.135	0.111	0.104	0.196	0.207	0.122

Values in parentheses represent robust standard errors. * represents *p* < 0.05, ** represents *p* < 0.01, and *** represents *p* < 0.001.

In terms of urban–rural differences, physical exercise exerted a significantly stronger positive influence on the reemployment of rural older adults than on their urban counterparts. After retirement, rural older adults typically find reemployment in physically demanding roles such as farm work, livestock assistance, and construction labor, which demand higher levels of endurance, strength, and flexibility. Systematic physical exercise can effectively improve cardiopulmonary function and decelerate muscle loss, thereby directly extending their working lifespan. In contrast, urban older adults tend to seek reemployment in light-duty or mentally demanding roles, such as community services and organizing cultural activities, where physiological demands are relatively lower. Consequently, the marginal benefits of physical exercise are limited, making it difficult to effectively translate the benefits of exercise into employment competitiveness. This finding suggested that the promoting effect of physical exercise on reemployment tended to be positively associated with the physical demand intensity of reemployment occupations, and the health capital accumulated through exercise was more likely to be converted into employment advantages in labor-intensive jobs.

In terms of area differences, physical exercise played a more significant role in promoting reemployment among older adults in the eastern regions than in the central and western regions. The developed service sector and flexible job market in the east have created a large number of positions suitable for older adults (such as supermarket stocking, domestic services, and community elder care), which require a certain level of physical fitness and flexible working schedules. In contrast, due to their more traditional industrial structures and limited reemployment opportunities, older adults in the central and western regions tend to be concentrated in low-income self-employment or domestic work, making it difficult to realize the economic benefits of physical exercise. In addition, the well-developed community sports facilities and older adults education systems in the eastern regions provide strong support, whereas the scarcity of sports resources in rural areas of the central and western regions limits the effectiveness of exercise. This indicated that the economic value of physical exercise for older adults reemployment was to some extent dependent on the regional economic structure and the availability of age-friendly employment opportunities, and the conversion of health capital to employment capital often required a sound external labor market environment as an important support.

In terms of age category differences, physical exercise had a more significant effect on promoting reemployment among younger older adults aged 60–70 years than among those aged 71 years and above. Since physical functional deterioration was less severe in the ages of 60–70, moderate-intensity exercises such as brisk walking and swimming can effectively improve their muscle strength and balance, meeting the physical demands of jobs such as parcel sorting and property maintenance. At the same time, this group was undergoing a transition in their social roles and had a strong desire to reenter the workforce. In addition to improving physical fitness, physical exercise could enhance their self-confidence and sense of social participation, indirectly facilitating access to employment opportunities. In contrast, reemployment for those aged 71 years and above primarily focused on low-intensity positions such as light manual labor and home-based contract work. The natural decline in physical functions significantly reduced the marginal benefits of physical exercise for this group. This suggested that the early retirement stage (60–70 years old) was the main period when physical exercise exerts a relatively strong effect on older adults’ reemployment, and interventions targeting this group were likely to achieve higher return on investment in human resource development.

In terms of educational level, physical exercise played a slightly greater role in promoting reemployment among older adults with a junior middle school education or above compared to those with lower levels of education. The former group was more likely to access scientific exercise knowledge through channels such as community lectures and online courses, enabling them to select activities suited to their needs, such as brisk walking or tai chi, thereby enhancing the systematic nature and effectiveness of their exercise routines. At the same time, they were more inclined to pursue positions requiring communication skills and a service-oriented mindset (such as supermarket sales associates or older adults education assistants). The vigorous appearance and good physical fitness cultivated through exercise enhance their competitiveness in the job market. In contrast, older adults with lower education often perceived exercise as merely routine physical labor due to limited health awareness. Their lack of scientific planning diminished the empowering effects of physical exercise on reemployment. Their reemployment opportunities were typically concentrated in entry-level positions, where limited technical requirements result in modest increases in economic returns. This reflected that educational level might moderate the effect of physical exercise on reemployment by influencing both the quality of exercise behavior and the type of reemployment positions, and improving health literacy was an important basis for better exerting the employment benefits of exercise.

In terms of occupation before retirement, physical exercise plays a significantly greater role in promoting reemployment among older adults who were ordinary workers before retirement than among those who were entry-level managers or held higher-level positions. After retirement, ordinary employees commonly take on physically demanding jobs such as community sanitation or supermarket stocking, which have clear requirements for continuous working hours and the ability to carry heavy loads. Regular exercise can effectively extend their working hours and reduce the risk of workplace injuries. In contrast, the reemployment options for grassroots managers and those in higher-level positions, such as industry consultants or corporate supervisors, primarily involved knowledge-intensive roles that rely on industry experience, professional networks, and decision-making skills. The physical fitness gains from exercise may not be a core competitive advantage for their reemployment. This demonstrated that the core competitive factors for older adults’ reemployment varied with pre-retirement occupational backgrounds, and physical exercise tended to be more effective for groups whose reemployment competitiveness depends more on physical capital rather than human capital or social capital.

## 5 Discussion

### 5.1 Theoretical implications: theoretical implications of mechanisms and heterogeneity

This study empirically examined the significant promotional effect of physical exercise on reemployment among older adults and validated three key mediating pathways: physical health, cognitive function, and social participation. Rooted in human capital theory and social capital theory, this study constructed a multi-dimensional analytical framework for the impact of physical exercise on older adults’ reemployment. These findings provided new empirical evidence for understanding the interactive relationship between physical health and labor force participation among older adults within the framework of “active aging.” From a human capital perspective, physical exercise was associated with improved physiological and cognitive capital of older adults, which might be attributed to its role in slowing the decline of physiological functions, strengthening cardiopulmonary function, and improving executive function and memory. This provided a sustainable physical and intellectual foundation for their re-entry into the labor market, reflecting the current trend in the new economic landscape where older adults’ employability increasingly relies on “soft skills” rather than mere physical strength. This was consistent with the core proposition of human capital theory that health was an important component of human capital, and investment in health could improve labor productivity and extend the working life cycle.

More notably, social participation, as a key mediating mechanism through which physical exercise influences reemployment among older adults, highlighted the role of social capital theory in understanding older adults’ reemployment. Physical exercise referred not only to individual physical activity but also to a process of social interaction, network building, and role reconstruction. Through group exercise and community activities, older adults rebuilt social trust, obtained employment information, and strengthened social identity, thereby effectively overcoming age discrimination and information barriers and achieving the transition from “retirees” to “re-employed individuals.” This mechanism aligns deeply with the “harmonious coexistence” characteristic of China’s social structure, revealing that while physical exercise promotes the multidimensional development of capabilities among older adults, it simultaneously facilitates the tangible construction of structural social capital and the conceptual reshaping of cognitive social capital through the optimization and reorganization of social support networks. This verified the core view of social capital theory that social networks and social trust could reduce information asymmetry and transaction costs, thereby improving the efficiency of labor market matching.

Furthermore, the study found that the empowering effects of physical exercise vary significantly across urban–rural regions, areas, age groups, educational levels, and occupational backgrounds. This suggested that the impact of physical exercise on reemployment was actually the result of a complex interplay among individual resource endowments, the structure of social opportunities, and life-stage characteristics. For example, differences between rural and urban older adults individuals in terms of employment types, physical demands, and social support systems determined that the marginal utility of physical exercise varies significantly. The contrast between the developed service economy in the eastern regions and the traditional industrial structure in the central and western regions reflected the moderating role of macroeconomic structures on individual health behaviors and employment outcomes. These differences suggested that the association between physical exercise and reemployment among older adults exhibited significant context-dependence, with its mechanism of action essentially being a dynamic coupling process involving individual health capital, social opportunity structures, and life-cycle characteristics. This necessitates that, when formulating relevant policies, policymakers accurately identify the bottlenecks and leverage points in the conversion of health capital across different groups to facilitate an effective transfer of value from physical exercise as an individual health behavior to a social employment resource.

### 5.2 Practical implications: dual empowerment in policy coordination and cultural transformation

As the population aging trend accelerated and the labor force underwent structural transformation, the practical value and policy implications of this study’s findings provide empirical support for building a “physical and health integration” framework with Chinese characteristics. Currently, China is at a critical stage of transitioning from “older adults’ care” to “supporting the older adults” and leveraging the potential of the older adults. Relying solely on the social security system was no longer sufficient to address the comprehensive challenges posed by an aging population. Physical exercise, as a low-cost, high-return, and easily scalable health intervention, possesses significant positive externalities and should serve as a strategic point for developing the human resources of older adults and revitalizing the “silver economy.” The government urgently needs to promote cross-departmental coordination and policy empowerment among the sports, health, and human resources systems. Specifically, drawing on the experience of “sports-health integration” pilot programs, an integrated service platform for “health assessment—exercise prescription—employment referral” should be established at the community level, with a focus on providing support to rural and resource-poor areas in central and western China to bridge the digital divide and resource gaps. Meanwhile, it is essential to leverage innovative platforms, such as the sports campus of the National University for the Older adults, to develop comprehensive curricula that combine physical skills, social skills, and job training, thereby helping older adults transition from exercise to employment. Such policies not only offer economic benefits, such as alleviating pressure on pension expenditures, expanding the labor supply, and stimulating new consumer markets, but also contribute to social governance by extending older adults’ healthy life expectancy and their period of social contribution, thereby enhancing their sense of fulfillment, happiness, and security.

In traditional Chinese perspectives on older adults’ care, older adults are often viewed as passive recipients of support and care, and the value and willingness of their continued participation in economic and social life have long been underestimated. This study confirmed that physical exercise, by enhancing physical fitness, expanding social networks, and boosting self-confidence, can effectively help older adults overcome both the internalization of age discrimination and external structural barriers, thereby regaining social recognition and achieving self-actualization. This represented a cognitive shift from passive aging to active aging. Therefore, governments, businesses, and social organizations should join forces to foster a social consensus and an inclusive culture that embraces the concept of active aging. Businesses can incorporate the benefits of physical exercise into employee health management and career evaluation systems, establish older adults-friendly positions and flexible work arrangements, and increase their willingness to hire older workers; the media should actively promote success cases of older adults reintegrating into society through exercise to challenge public stereotypes. Only when physical exercise and reemployment are no longer viewed as isolated phenomena, but are widely recognized as vital pathways for older adults to realize their intrinsic value and continue contributing to society, can China truly achieve a successful transition from the “era of longevity” to the “era of health” and the “era of contribution,” thereby offering a solution to the global challenge of population aging that is both wise and uniquely Chinese.

### 5.3 Limitations

This study has several limitations. First, this study used cross-sectional data from the 2023 CLASS survey, which could only reveal the correlation between variables but could not establish a definitive causal relationship. Although we used propensity score matching to alleviate selection bias caused by observable variables, unobservable confounding variables such as pre-retirement career motivation, personality traits (e.g., conscientiousness), and genetic endowment may simultaneously affect physical exercise habits and reemployment willingness, leading to potential omitted variable bias.

Second, all variables in this study were measured based on self-reported data from the respondents, which might be subject to recall bias and social desirability bias. For example, respondents might overreport their physical exercise frequency or underreport their health problems. In addition, the measurement of physical exercise only considered frequency but not intensity, duration and type, which might mask the dose–response relationship between exercise and reemployment.

Third, the measurement of social participation mainly focuses on formal community activities, but did not include informal social interactions such as family gatherings and neighborhood exchanges, which might underestimate the actual level of social participation of the older adults. Similarly, the measurement of reemployment only considered paid work but did not include unpaid productive activities such as family labor and volunteer work, which limited the generalizability of the findings.

Finally, the total mediation effect of the three pathways accounted for only about 26.5% of the total effect (10.3% + 4.9% + 11.3%), meaning that approximately 73.5% of the total effect remained unexplained by the mechanisms examined in this study. This indicated that the direct effect of physical exercise on older adults’ reemployment was still substantial, and there might be other important mediating pathways that had not been identified. In addition, all reported mediation proportions were point estimates from the sample, which had inherent sampling uncertainty. The confidence intervals of the mediation proportions for different pathways may overlap, so the minor differences in point estimates should not be overinterpreted as absolute differences in the strength of the mediating effects. This further suggested that physical exercise might affect older adults reemployment through other mechanisms not explored in this study, such as mental health, self-efficacy, and life satisfaction, which needed to be systematically examined in future longitudinal research.

## 6 Conclusion and suggestions

### 6.1 Conclusion

First, physical exercise had a stable and significant positive correlation with reemployment among older adults, and this association had been verified to be robust through multiple methods including propensity score matching and alternative model specifications. Second, this association was mediated by three parallel pathways: physical health, cognitive function, and social participation, with social participation being the most critical mediating mechanism. Third, the strength of this association exhibited significant population heterogeneity: it was more pronounced among rural older adults, those in eastern regions, those aged 60–70 years, those with junior middle school education or above, and those who were ordinary workers or clerks before retirement.

### 6.2 Suggestions

(1) Strengthen policy coordination and establish a “support network” for the integration of sports and public health. Integrate policies on aging, sports development, and employment services, and incorporate physical exercise into the reemployment support system for older adults. On the one hand, increase the allocation of sports resources to rural and central-western regions, improve community fitness facilities suitable for older adults, and narrow the urban–rural gap. On the other hand, establish a mechanism for integrating sports and public health, combining health screenings for older adults with scientific exercise guidance, and implementing an “exercise prescription + employment assessment” system. This will precisely align health improvement with reemployment needs, making physical exercise a key lever for developing the human resources of older adults. Specifically, community health service institutions can integrate physical fitness assessment into the regular health check-ups for older adults, and issue personalized exercise prescriptions combined with their physical conditions and reemployment intentions. At the same time, establish a linkage mechanism between community sports service centers and human resource service agencies to provide targeted employment referral services for older adults with good physical conditions and reemployment willingness.
(2) Optimize the supply of older adults-friendly services and establish a bridge between physical exercise and employment. To address the needs of older adults in aligning exercise with employment opportunities, provide tiered and categorized services: for rural older adults who prefer physically demanding jobs, offer targeted training in endurance and strength; for younger urban older adults, design sports activities that incorporate social interaction, while simultaneously integrating information on job opportunities such as community services and retail sales. At the same time, leveraging the sports branches of the National University for Older Adults, develop integrated exercise skills and employment training programs. These programs will combine exercise forms such as tai chi and square dancing with career paths in older adults’ education and community volunteer services, facilitating a seamless transition from fitness to employment. For example, we can cultivate outstanding sports for older adults enthusiasts as community sports instructors and health promoters, and develop positions such as activity organizers in universities for older adults and community activity centers that match the skills and characteristics of older adults.
(3) Precisely activate group potential and develop a road for targeted measures. Formulate differentiated strategies based on group characteristics: for younger older adults aged 60–70, promote moderate-intensity exercise to maintain physical fitness and connect them with positions such as parcel sorting and property maintenance; provide scientific exercise guidance to older adults with a junior middle school education or above to enhance their competitiveness in service-oriented roles; and for retired employees, extend their ability to perform physical labor through regular exercise and reduce the risk of workplace injuries upon reemployment. At the same time, encourage businesses to incorporate the outcomes of physical exercise into the performance evaluation system for older employees, fostering a virtuous cycle where exercising promotes employment and employment reinforces exercise, thereby effectively advancing the goal of “active aging.”

## Data Availability

The original contributions presented in the study are included in the article/supplementary material, further inquiries can be directed to the corresponding author.
